# Diet, Fecal Microbiome, and Trimethylamine N-Oxide in a Cohort of Metabolically Healthy United States Adults

**DOI:** 10.3390/nu14071376

**Published:** 2022-03-25

**Authors:** Kristen L. James, Erik R. Gertz, Eduardo Cervantes, Ellen L. Bonnel, Charles B. Stephensen, Mary E. Kable, Brian J. Bennett

**Affiliations:** 1 Department of Nutrition, University of California-Davis, One Shields Avenue, Davis, CA 95616, USA; krijames@ucdavis.edu (K.L.J.); ecervantes@ucdavis.edu (E.C.); ellen.bonnel@usda.gov (E.L.B.); charles.stephensen@usda.gov (C.B.S.); mary.kable@usda.gov (M.E.K.); 2 USDA-ARS Western Human Nutrition Research Center, 430 West Health Sciences Drive, Davis, CA 95616, USA; erik.gertz@usda.gov

**Keywords:** trimethylamine n-oxide, TMAO, ASA24, microbiome, TNF-alpha, inflammation, endoPAT, endothelial function

## Abstract

TMAO is elevated in individuals with cardiometabolic diseases, but it is unknown whether the metabolite is a biomarker of concern in healthy individuals. We conducted a cross-sectional study in metabolically healthy adults aged 18–66 years with BMI 18–44 kg/m^2^ and assessed the relationship between TMAO and diet, the fecal microbiome, and cardiometabolic risk factors. TMAO was measured in fasted plasma samples by liquid chromatography mass spectrometry. The fecal microbiome was assessed by 16S ribosomal RNA sequencing and recent food intake was captured by multiple ASA24 dietary recalls. Endothelial function was assessed via EndoPAT. Descriptive statistics were computed by fasting plasma TMAO tertiles and evaluated by ANOVA and Tukey’s post-hoc test. Multiple linear regression was used to assess the relationship between plasma TMAO and dietary food intake and metabolic health parameters. TMAO concentrations were not associated with average intake of animal protein foods, fruits, vegetables, dairy, or grains. TMAO was related to the fecal microbiome and the genera *Butyribrio*, *Roseburia*, *Coprobaciullus*, and *Catenibacterium* were enriched in individuals in the lowest versus the highest TMAO tertile. TMAO was positively associated with α-diversity and compositional differences were identified between groups. TMAO was not associated with classic cardiovascular risk factors in the healthy cohort. Similarly, endothelial function was not related to fasting TMAO, whereas the inflammatory marker TNF-α was significantly associated. Fasting plasma TMAO may not be a metabolite of concern in generally healthy adults unmedicated for chronic disease. Prospective studies in healthy individuals are necessary.

## 1. Introduction

Cardiovascular diseases (CVDs) are the leading causes of death worldwide and account for one in three deaths in the United States alone [1,2]. Classic CVD risk factors include high blood pressure, hyperlipidemia, smoking, diabetes, obesity, and lack of physical activity; however, advances in multiomic technologies have offered unbiased and unprecedented insights to the biological mechanisms of disease and have revealed new CVD risk factors such as the plasma metabolite trimethylamine n-oxide (TMAO). TMAO can be obtained in two ways including the direct consumption of TMAO containing foods and the meta-organismal metabolism of quaternary amines [3]. In the latter method, unabsorbed dietary nutrients including choline, carnitine, phosphatidylcholine, and betaine are utilized by the gut microbiota to generate trimethylamine (TMA), which is absorbed and oxidized in the liver by flavin-containing monooxygenase 3 (FMO3) to TMAO [4,5]. TMAO is robustly associated with CVD events and death in prospective studies conducted in metabolically unhealthy individuals; however, it is unclear whether TMAO is a relevant risk factor in healthy individuals [6,7]. Furthermore, inter- and intra-variation in TMAO concentrations have been reported suggesting the regulation of TMAO is complex [8,9]. For example, the canonical TMAO pathway, defined by diet, the gut microbiome, hepatic oxidation, and excretion contains modifiable (diet), non-modifiable (FMO3 genotype, kidney function), and semi-modifiable factors (the gut microbiome), introducing multiple levels of variability. It is unknown whether manipulating the diet, or the gut microbiome will alter TMAO concentrations and if the responses are dependent on factors such as sex, age, BMI, or health status.

Consuming eggs, meat, and the tissue of deep-sea fish are commonly associated with plasma TMAO due to their levels of phosphatidylcholine, carnitine, and TMAO, respectively [3,10,11]. Although feeding studies have clearly demonstrated a post-prandial rise in TMAO after consumption of these foods or nutrients, the long-term effect on fasting concentrations is unclear. It is probable that both the post-prandial and long-term effects on TMAO concentrations are dependent, in part, on the metabolite and its form. Eggs are a nutrient-dense food with yolks abundant in the lipid phosphatidylcholine and have consistently demonstrated null effects on fasting TMAO concentrations. For instance, a randomized-controlled feeding trial providing eggs or egg whites found no difference in fasting TMAO concentrations after four weeks [12]. In a randomized controlled feeding trial, which compared the effects of two eggs per day versus oatmeal, no significant differences in TMAO concentrations by breakfast food were observed [13]. Red and white meat contain choline and carnitine mechanistically linking them to TMAO. A randomized two-arm cross-over design investigating the effect of red versus white meat observed effects of red (choline, 572 mg/d; carnitine, 258 mg/d) but not white meat (choline, 498 mg/d; carnitine, 56 mg/d) on TMAO after four weeks of consumption [10].

The effect of individual foods on TMAO concentrations may vary due to a person’s overall diet pattern. Considering the fiber and carbohydrate content of the diet, a randomized two-arm cross-over feeding study providing low- or high-levels of resistant starch on either a low- or high-carbohydrate background diet resulted in increased TMAO concentrations with the high-resistant starch, low-carbohydrate diet confirming a nutrient by diet pattern interaction [14]. Additionally, a Mediterranean diet pattern with 200 versus 500 g of unprocessed lean red meat per week identified lower TMAO concentrations on the 200 but not the 500 g diet, suggesting that reducing red meat is an effective strategy for lowering TMAO [15]. Evidently, the nutrient form, the food matrix, and the diet pattern affect TMAO but how these dynamics play out in a free-living cohort has not been well documented.

The relationships among nutrients, foods, diet patterns, and TMAO may be due to the impact of each on the gut microbiome, which has been clearly implicated in the generation of TMAO. This has been demonstrated in rodents and humans such that antibiotics negate the effect of choline or carnitine feeding on postprandial TMAO concentrations and in humans such that, individuals consuming a vegan diet produced a reduced postprandial TMAO response after a carnitine meal challenge than matched omnivore controls [4,5,16]. Furthermore, metagenomic screenings have identified TMA-generating gene clusters, which has led to the identification that, commonly, *Firmicutes* but not *Bacteroidetes* contain the TMA-generating gene cluster choline utilizing C and glycyl radical activating protein (CutC/D) [17,18]. Gene clusters that utilize carnitine or its degradation product γ-butyrobetaine have also been characterized including a two-component Rieske-type oxygenase/reductase (CntA/B) that is commonly observed in *Proteobacteria* [19,20]. Although gene clusters have been identified, a metagenome analysis failed to predict TMAO concentrations from TMA-generating genes in a cohort of 113 adults. This result suggests that other TMA-generating genes have yet to be discovered, that TMAO-reducing genes may be important, that the fecal microbiome provides a poor representation of TMAO dynamics, or that the gut microbiome’s role in regulating fasting TMAO concentrations is important but minor [21]. To better understand which mechanistic steps drive fasting TMAO concentrations, studies need to consider diet, the gut microbiome, and host health in tandem. Additionally, it is unknown whether differences in the gut microbiomes of generally healthy adults relate to TMAO concentrations.

The objectives of our study are to assess the biological role of TMAO in a generally healthy cohort of adults living in the United States by characterizing the concentrations of TMAO and relating these concentrations to recent diet intake, the fecal microbiome, and cardiometabolic risk factors. Notably, our study utilizes data from a cross-sectional study equally stratified by sex, age, and BMI, allowing us to probe how these characteristics affect our outcomes.

## 2. Materials and Methods

### 2.1. Study Participants

The Nutritional Phenotyping Study was conducted at the USDA-Agriculture Research Service, Western Human Nutrition Research Center (WHNRC) from June 2015 to July 2019. Participants were recruited from Davis, CA, and the surrounding area. The study used a binning approach to equally recruit participants by sex (male and female), age (18–33 years, 34–49 years, and 50–65 years), and BMI (18.5–24.99, 25–29.9, and 30–44 kg/m^2^), resulting in equal stratification of 393 participants into 18 bins. Of these subjects, 361 participants had complete plasma chemistry, 356 had at least two complete dietary recalls, and 355 had 16S rRNA bacterial gene sequencing from the fecal microbiome (Supplemental Figure S1).

The inclusion and exclusion criteria and the study design have been previously described [22]. Briefly, exclusion criteria included pregnancy, lactation, egg allergy, recent minor surgery or major surgery within the last 16 weeks, recent antibiotic treatment, hospitalization within the past 4 weeks, systolic blood pressure over 140 mmHg, or taking daily medication for a chronic disease including, but not limited to: diabetes mellitus, cardiovascular disease, cancer, gastrointestinal disorders, kidney disease, liver disease, bleeding disorders, asthma, autoimmune disorders, hypertension, and osteoporosis, or use of prescription medications at the time of the study that directly affected the primary study’s endpoints (e.g., hyperlipidemia, glycemic control, steroids, statins, anti-inflammatory agents, and over-the-counter weight loss aids). The strict exclusion criteria resulted in recruiting generally healthy participants. Ethical approval for the study was received from the University of California, Davis, Institutional Review Board (691654) and written informed consent was obtained in compliance with the IRB guidelines for participation in the study.

### 2.2. Study Design

The study consisted of two visits to the WHNRC approximately two weeks apart. In the first visit, the participant’s sex-age-BMI bin was confirmed, and they were trained to collect bio-samples and to complete a diet recall using the Automated Self-Administered 24-h Dietary Assessment Recall System (ASA24). Between visits, participants were randomly prompted to complete two weekday and one weekend ASA24 diet recalls (ASA24, versions 2014 and 2016) to capture recent dietary intake. The evening prior to their second visit, participants consumed a standardized meal provided by the study and completed a 12-h overnight fast. The food composition of the standardized meal is provided in Supplemental Table S1 and the nutrient composition in Supplemental Table S2. The meal contained 280.7 mg of choline and 2.4 mg of betaine. Participants were instructed to collect a stool sample using provided collection supplies as close to their second visit as possible. Stool samples were kept on ice packs and processed the day of transport. Notable to the present study, upon the participant’s second visit, a fasted blood draw was collected, and endothelial and arterial health was measured using the EndoPAT system following the manufacturer’s instructions (Itamar Medical, Caesarea, Israel).

### 2.3. Dietary Assessment

The ASA24 recalls were rigorously quality checked by a registered dietitian following National Cancer Institute (NCI) guidelines and only participants with at least two recalls were included [23,24]. The recalls of each participant were averaged to represent average daily food intake. Due to individuals eating foods which contain nutrients, and due to the relevance of fish, meat, and eggs to the TMAO pathway, we focused our study on ASA24 variables that represent the total intake of food groups. Variables were assessed for compliance to the normal distribution by the Shapiro–Wilk test W-statistic. Variables were considered normal if their W-statistic was greater than 0.95. Variables with a W-statistic less than 0.95 were first tested via natural log transformation and then square root. Food groups with low consumption including seafood, soy, and yogurt were turned into binary “does consume” or “does not consume”. Organ-meat food categories were ubiquitously non-consumed and were removed. We also included three miscellaneous diet markers including the Healthy Eating Index (HEI) total score, total choline intake, and total fiber intake. The HEI is a measure of diet quality related to the Dietary Guidelines for Americans and was calculated following the guidelines of the NCI [25]. Total choline and total fiber are variables provided by the ASA24.

### 2.4. Quantification of TMAO and Select Amines

TMAO, choline, carnitine, and betaine were measured in plasma from 361 fasted participants using liquid chromatography-high resolution mass spectrometry (LCMS) using a method adapted from Wang and colleagues [4]. Briefly, 20 μL of plasma was extracted and added to 80 μL of 10 μM surrogate standard consisting of deuterated analytes in 1:1 acetonitrile:water (Sigma-Aldric, St. Louis, MO, USA). Samples were vortexed for 30 s and centrifuged at 18,000 g for 10 min at 4 °C. Standards ranging from 0 to 100 μM of non-deuterated analytes in 1:1 acetonitrile:water in methanol were used to establish a standard curve. Quantification of TMAO was performed by LCMS using a silica column (4.6 by 250 mm, 5 μm Luna silica; Cat. No. 00G-4274-E0, Phenomenex, Torrance, CA, USA) at a flow rate of 0.8 mL/min using a Waters Acquity UPLC (Waters, Milford, MA, USA). A discontinuous gradient of solvent A (0.1% propionic acid in water) and solvent B (0.1% acetic acid in methanol) beginning at 2% B linearly to 15% B over 10 min, then linearly to 100% B over 2.5 min, held for 3 min, and returning to 2% B. Analytes were monitored using electrospray ionization in positive-ion mode with multiple reaction monitoring of precursor and characteristic production transitions. Precision and accuracy were assessed by employing triplicates of two different human plasma sample controls in each batch of approximately 45 samples. Analyst software (Sciex, version 1.6.2, Redwood City, CA, USA) was used for the integration and quantification of the analytes.

### 2.5. Clinical Chemistry

Clinical chemistry was measured on the COBAS INTEGRA 400 Plus analyzer (Roche Diagnostic Systems, Basel, Switzerland) following the manufacturer’s instructions. Human blood was processed to plasma upon collection and was stored at −80 °C until analysis.

### 2.6. Inflammatory Markers

Fasting concentrations of the inflammatory markers tumor necrosis factor alpha (TNF-α), interleukin 6 (IL-6), and c-reactive protein (CRP) were assayed in EDTA plasma using an electrochemiluminescence-based detection platform with multiplexed immunoassays using the V-PLEX system according to the manufacturers specifications (Mesoscale Discovery, LLC). CRP was measured using the V-PLEX Vascular Injury Panel 1 with samples diluted 1:1000. TNF-α and IL-6 were measured using the V-PLEX Custom Human Biomarker Proinflammatory Panel 1 with samples diluted 1:2. Three levels of lyophilized controls were used on each plate to assess plate to plate variation and mean concentrations of duplicate wells were used for analysis.

### 2.7. Microbiota Assessment

Detailed methodology of the stool sample collection, bacterial DNA extraction, and gene sequence analysis has been published [26]. Briefly, samples were collected using provided sanitary collection supplies and stored on ice in a hard-sided cooler for transport to the research center between 1 and 24 h. Samples were homogenized, rolled to a uniform thickness, scored into 1 cm ribbons, and stored at −80 °F [26]. Bacterial DNA was isolated using the ZymoBiomics kit (ZYMO Research, Irvine, CA, USA) from homogenized stool samples collected within 3 days of the participant’s second visit. Samples were sent to Dalhousie University for the library preparation and sequencing [27]. Prior to sequencing, the 16S rRNA V4-V5 hypervariable region sequences were amplified and barcoded using the universal (515F (Parada) and 926R (Quince)) primers [28,29]. Amplicons were verified via gel electrophoresis, cleaned, multiplexed, and sequenced via the Illumina MiSeq using 2 × 300 bp paired-end v3 chemistry (Illumina, San Diego, CA, USA).

The 16S sequencing data were processed using Qiime2 (v2019.10) [30]. Sequences were demultiplexed and trimmed using Cutadapt [31]. Sequence variants were identified using DADA2 and classified by the Naive Bayes classifier using the GreenGenes database (v13_8) at the threshold of 99% pairwise identity. The Qiime2 output was imported to RStudio using the packages qiime2R (v0.99.12), phyloseq (v1.26.1), and vegan (v2.5-6). Sequences were retained if they were present in 5% of samples. Shannon, Pielou’s evenness, and observed OTUs were used to describe sample alpha diversity. Dissimilarity matrices were calculated using the phyloseq distance function and multivariate homogeneity of variances was tested using the PERMDISP2 method via the betadisper function. Analysis of variance using distance matrices was tested via adonis2 with 999 permutations and controlled for sex, age, and BMI. The ordinate function was employed to conduct principal coordinate analysis. Differentially abundant microbes were identified using the log likelihood test comparing the full and null models with the covariates sex, age, and BMI via DESeq2 (v1.32.0) [32].

### 2.8. Endothelial Function with EndoPAT

Endothelial function was assessed using the EndoPAT system following the manufacturer’s instructions. Prior to the evaluation, participants lay in a supine position for 15 min and were instructed to remain still. The occlusion was performed on the non-dominant arm and lasted 5 min at 200 mmHg followed by 5 min post-occlusion. The reactive hyperemia index (RHI) and augmentation index (AI) were used to assess endothelial function and arterial stiffness, respectively. AI is described in Table 1; however, the augmentation index normalized to a heart rate of 75 beats per minute (AI75) was used in the linear regression analysis to comply to parametric assumptions.

### 2.9. Statistical Analysis

All analyses were completed in R Studio (v 4.1.0, Boston, MA, USA). All diet variables and metabolic health parameters were assessed for compliance to the normal distribution by the Shapiro–Wilk test. Variables with a W-statistic greater than 0.95 were considered normal. Non-compliant variables were first tested by the natural log transformation and then square root. The participants were ranked by their TMAO concentration and cut into three equal number groups creating TMAO tertiles. Descriptive statistics were computed by fasting plasma TMAO tertiles and evaluated by ANOVA and Tukey’s post-hoc test. Statistics are represented as mean ± standard deviation unless otherwise noted. Multiple linear regression was used to assess the relationships between plasma TMAO and dietary food intake and metabolic health parameters. Covariates were identified if they had a significant relationship with fasting plasma TMAO and were supported by the literature. Of the sex, age, and BMI criteria used in recruitment, a significant sex by age interaction was identified (*p* = 0.026) and used as a covariate (Supplemental Figure S2). In the diet analysis, TMAO was the response variable, and the models were adjusted for a sex by age interaction, fasting plasma cystatin C—a validated marker of kidney function, and total energy intake. In the metabolic health analysis, TMAO acted as an explanatory variable and the models were adjusted for sex, age, and fasting plasma cystatin C. Analyses were corrected for multiple testing via the Benjamini and Hochberg method and significance was set at Padj < 0.05.

## 3. Results

### 3.1. Participant Characteristics

Female (*n* = 189) and male (*n* = 172) participants were recruited across the spectra of ages and BMIs and ranged in age from 18 to 66 years and in BMI from 18 to 44 kg/m^2^. Descriptive characteristics of the participants by TMAO tertile is provided in Table 1 and demonstrate that the cohort was generally healthy with parameters within acceptable clinical ranges. The multi-ethnic cohort was mostly represented by Caucasians followed by Hispanics and Asians (Table 1). Fasting TMAO concentrations were significantly lower in Asians compared to Hispanics and Caucasians, but no significant effect of ethnicity on TMAO concentrations was identified after correcting for a sex by age interaction and fasting plasma cystatin C.

Fasting plasma TMAO concentrations ranged from 0.44 to 22.5 μM with a median of 3.32 μM. TMAO was broken into three tertiles; the low tertile had a mean of 2.05 ± 0.55 (range 0.44 to 2.77 μM), the middle tertile had a mean of 3.35 ± 0.32 (range 2.78 to 3.9 μM), and the high tertile had a mean of 6.18 ± 3.11 (range 3.91 to 22.5 μM). The tertiles were equally represented by sex and BMI, but increased in age (*p* = 0.002). Fasting plasma choline concentrations but not betaine nor carnitine significantly increased with TMAO tertile (*p* = 0.024).

### 3.2. TMAO and Recent Food Intake

To understand how diet may be contributing to TMAO concentrations, we described the recent intake of foods and assessed the relationship between foods, nutrients, and HEI total score to fasting TMAO. The participants averaged 1.63 ± 2.08 oz. equivalents of red meat from beef, veal, pork, lamb, or game, 0.93 ± 1.36 oz. equivalents of processed or cured meat from frankfurters, sausages, corned beef, or luncheon meat, and 1.80 ± 2.34 oz. equivalents of poultry per day. The average intake of eggs was 0.71 ± 0.72 oz. equivalents, which represents less than one egg per day. For both females and males, the average intake of choline from foods and supplements was below the adequate intake (choline adequate intake, 425 mg/day in females and 550 mg/day in males) at 332.70 ± 121.72 mg for females and 461.1 ± 207.90 mg for males.

Overall, the intake of foods did not differ by TMAO tertile (Supplemental Table S3). This included the intake of foods abundant in choline and carnitine such as animal protein foods, as well as vegetables and grains. The HEI total score, which is a measure of diet quality related to the Dietary Guidelines for Americans was not different by tertile and averaged 61.87 out of 100 (range 22.47 to 94.33, standard deviation 13.15). When the relationship between food and TMAO was evaluated while controlling for covariates (sex, age, fasting plasma cystatin C, and average total calorie intake), no significant relationships were identified (Table 2).

### 3.3. Fecal Microbiome Relates to TMAO

**Figure 1:**
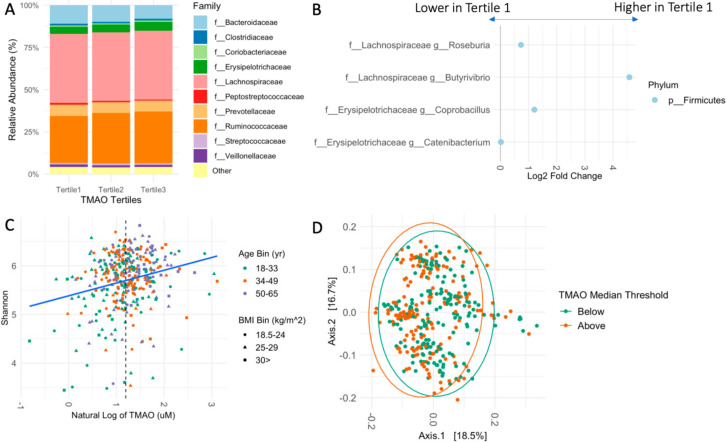
Fecal microbiome relates to fasting plasma TMAO in a generally healthy cohort. (**A**) Relative abundance of the top 10 most abundant families by TMAO tertile. (**B**) Genera that are differentially abundant between the low (TMAO 0.44–2.77 μM) and high TMAO tertile (TMAO > 3.91 μM). Graph is relative to the low group such that points to the right of 0 are more abundant in the low tertile. (**C**) Alpha diversity is directly related to fasting plasma TMAO (Shannon diversity, β = 0.17, *p* < 0.001). Point colors represent age bin and shape represents BMI bin. (**D**) Compositional differences are identified between individuals with TMAO less than and greater than 3.32 μM (Unweighted UniFrac, *p* = 0.001).

We identified 2786 amplicon sequence variants (ASVs) in the cohort, which were represented by seven phyla and 52 genera (Supplemental Table S4). Regardless of the participant’s TMAO-tertile, Firmicutes and Bacteroides were the dominant phyla and Lachnospiraceae, *Ruminococcaceae*, and *Bacteroidaceae* were the most abundant family (Figure 1A and Supplemental Table S5). Similarly, the two most abundant genera, *Blautia* and *Faecalibacterium*, were not impacted by TMAO classification. In the highest TMAO-tertile, *Ruminococcus* was the third most abundant genus, whereas Bacteroides held this position in the low and middle TMAO-tertiles (Supplemental Table S6). This finding was corroborated by the differential abundance analysis which identified that the lowest TMAO-tertile was enriched with the genera *Roseburia*, *Butyrivibrio*, *Coprobacillus*, and *Catenibacterium* compared to the highest tertile (Figure 1B and Supplemental Table S7). When the cohort was bisected by the median TMAO level of 3.32 μM, *Coprobacillus* and *Roseburia* remained more abundant in the low group. At the family level, participants in the highest tertile had elevated levels of *Christensenellaceae*, while participants in the lowest tertile had increased levels of *Bacteroidaceae* and *Peptostreptococcaceae* (Supplemental Figure S3 and Table S7), The Firmicutes to Bacteroidetes ratio increased from 3.48 to 4.43 to 4.76 per tertile.

Across measures of richness and/or evenness, alpha diversity was consistently associated with increased TMAO (Figure 1C). Assessed metrics include Shannon diversity (β = 0.17, *p* < 0.001), Faith’s phylogenetic diversity (β = 0.065, *p* < 0.001), Pielou’s evenness (β = 1.17, *p* = 0.01), and observed OTUs (β = 0.003, *p* < 0.001), suggesting the differences were due to which taxonomies were present and how many. Differences in the cohort’s overall composition were identified; unweighted UniFrac (*p* = 0.001), and Bray Curtis statistics (*p* = 0.017) were significantly different in participants with TMAO less than 3.32 μM versus participants with TMAO above that level (Figure 1D). Weighted UniFrac did not achieve statistical significance (*p* = 0.061).

### 3.4. Cardiometabolic Markers and TMAO

We assessed the relationship between fasting plasma TMAO and cardiometabolic risk factors including anthropometric, clinical chemistry, and systemic inflammation markers. TMAO was not related to BMI nor waist circumference (Table 3). Although TMAO has been mechanistically linked with atherosclerosis, we were unable to identify a relationship between fasting plasma TMAO concentrations and endothelial function by EndoPAT. Specifically, the reactive hyperemia index (RHI) showed no relationship to TMAO (β = −0.007, Padj = 0.963), nor was the augmentation index (AI) associated with circulating concentrations of fasting TMAO (β = −1.2, Padj = 0.630). Additionally, the classical blood cardiometabolic risk factor of glucose, insulin, or lipid levels were not associated with TMAO (Table 3).

TMAO was significantly correlated with TNF-α levels (β = 0.11, Padj = 0.024). We investigated whether the relationship between TMAO and TNF-α was driven by the gut microbiome; however, when the four differentially abundant taxa were included as covariates the relationship was unaffected (β = 0.11, Padj = 0.048). Furthermore, no relationships between the differentially abundant taxa and TNF-α were identified. Other markers of systemic inflammation, interleukin-6 (IL-6) and c-reactive protein (CRP) were not related to TMAO.

## 4. Discussion

TMAO has been associated with cardiovascular disease (CVD) in several studies, but studies of younger and generally healthy individuals have not always confirmed this association [4,5,33,34]. For example, TMAO was not associated with atherosclerosis measures in healthy early-middle-aged adults enrolled in the Coronary Artery Risk Development in Young Adults (CARDIA) study [34]. Furthermore, in a cohort of parents and children, TMAO was not related to metabolic syndrome nor markers of cardiovascular risk in either generation [35]. Thus, determining the relationships between TMAO and risk factors of CVD remain an important area of research prior to adopting dietary modifications to reduce TMAO concentrations. We sought to address this gap and to assess the drivers of TMAO variation by studying diet, the fecal microbiome, and cardiometabolic markers—known cornerstones of the TMAO pathway, in a generally healthy cohort of adults.

Plasma TMAO is primarily derived from the microbial metabolism of choline, phosphatidylcholine, and carnitine which are abundant in red and white meat, and eggs and are commonly consumed in the typical American diet. Feeding studies demonstrate that consuming foods abundant in the TMAO precursors will lead to a transient post-prandial rise in TMAO concentrations, but studies assessing fasting TMAO concentrations report mixed results [3,11,36,37]. To account for the intake of TMAO precursors as well as other dietary factors that may influence TMAO production, we assessed the relationship between recent dietary intake of animal-sourced foods, as well as vegetable, fruit, grain, and dairy food groups. We found no dietary associations between recent food intake and fasting metabolite concentrations in our healthy cohort. Although we did not observe a relationship between TMAO and meat intake, the cohort’s average intake of meat resembled national averages suggesting this was not due to reduced intake. For example, our cohort averaged 5.30 ± 4.18 oz. equivalent per day of animal sourced foods defined as the total of red and white meat, organ meat, seafood, and eggs, (males, 5.56 ± 4.96 oz. equivalent; females, 3.48 ± 2.85 oz. equivalent) compared to 2009–2010 What We Eat in America results showing males averaged 6.28 ± 0.13 oz. equivalent and females averaged 3.79 ± 0.099 oz. equivalent [38]. Considering habitual diet, the average HEI score for our cohort was 62 (0–100 point scale, higher scores reflect compliance to the Dietary Guidelines for Americans) compared to national averages of 56 for adults 19–30 years and 59 for adults 31–59 years (Supplemental Table S3) [39]. While consumption of meat was comparable to national averages, overall diet quality was more aligned to the Dietary Guidelines. This may have been driven by intakes of food groups such as whole grains, fruits, and vegetables, which may reduce TMAO concentrations. As such, it is possible that other dietary factors including the food matrix, or the timing of the meal play critical roles in the ability to generate TMAO from dietary constituents. Dietary patterns may shape the gut microbiome and TMAO concentrations, but we were unable to discern interpretable diet factors (dietary factor analysis data not shown). Overall, our findings support that recent food intake is not associated with TMAO concentrations in generally healthy individuals.

The gut microbiome plays an indispensable role in the metabolism of choline and carnitine to TMAO [4,5]. Therefore, we hypothesized that compositional differences would be identified between individuals with varying concentrations of TMAO. Measures of evenness and richness consistently revealed a direct relationship between α-diversity and TMAO. Alpha-diversity is commonly associated with healthy biological states making the direct relationship puzzling; however, accounts of TMAO concentrations increasing on healthy diets have been observed [40]. Further, fiber introduces complex oligosaccharides to support a diverse gut microbiome, yet total fiber intake did not differ between the TMAO tertiles suggesting other factors may drive the observed α-diversity relationship.

When subjects were classified as high or low responders of TMAO concentrations (i.e., below or above the median of 3.32 μM), compositional differences were identified. Additionally, we identified several differentially abundant taxonomies between the lowest (<2.18 μM) and highest TMAO tertile (>3.9 μM). The genus *Butyrivibrio* (Phylum Firmicutes, class Clostridia, order Clostridiales) is involved in butyrate production via the breakdown of plant polysaccharides and was more abundant in the low TMAO tertile. Although total fiber was not statistically different between the tertiles, diets of individuals in the first tertile may have been enriched in specific plant foods that promoted *Butyrivibrio* [41]. *Roseburia* (Phylum Firmicutes, class Clostridia, order Lachnospiraceae) is also a butyrate producer and was enriched in participants in the lowest tertile. In numerous strains of mice, *Roseburia* was negatively associated with atherosclerotic plaque size. Follow up fecal transfer experiments providing a core community with or without the strain *Roseburia intestinalis* demonstrated that mice given *Roseburia* had reduced levels of TNF-α and vascular cell adhesion molecule 1 [42]. Of the differentially abundant taxa, only the family Christensenellaceae (Phylum Firmicutes, class Clostridia, order Clostridiales) was more abundant in the highest tertile. Christensenellaceae has been reported to be highly heritable and widely associated with positive health states such as reduced adiposity [43]. Interestingly, Christensenellaceae had a strong inverse relationship to BMI (β = −3.8, *p* = 0.015); TMAO was not related to BMI (β = 0.829, Padj = 0.331). Relative to TMAO, we identified a stepwise increase in the Firmicute to Bacteroidetes ratio, a simplified marker generally associated with positive health states. We note that our resolution is limited to the genus level and that species within a genus may have different physiological effects.

Prospective cohort studies demonstrate that individuals with high baseline TMAO concentrations experience increased incidences of adverse cardiovascular events including heart attack and stroke [44]. Mechanistically, TMAO has been shown to increase platelet reactivity and thrombosis and has been linked to many inflammatory pathways including the NF-κβ cascade and increased expression of inflammatory cytokines and chemokines [45]. TNF-α is important in the activation of NF-κβ, which has been mechanistically linked with TMAO in mice, human aortic endothelial cells, and vascular smooth muscle cells. Additionally, TMAO may be linked to dysregulated glucose metabolism and altered cholesterol transport [46,47,48,49]. We assessed the relationship between TMAO and cardiometabolic markers in a healthy population and identified that TMAO was strongly associated with tumor necrosis factor-alpha (TNF-α). This observation replicates the finding of Rohrmann et al., who observed few relationships between diet and fasting TMAO but did observe a relationship between TMAO and inflammation [37]. Extending this finding, the significant relationship was unaffected when the abundances of the differentially abundant taxa were included as covariates suggesting that the inflammatory association was independent of the components of the gut microbiome related to TMAO. We tested whether we would be able to detect associations between TMAO and CVD risk factors within the clinically healthy range. We did not find associations between TMAO and the risk factors; however, CVD is an inflammatory disease that takes decades to develop, therefore, our TNF-α finding warrants further investigation.

Many reports including our own demonstrate that TMAO concentrations increase with age. Many CVD risk factors, and metabolic diseases increase with age making it difficult to ascertain the effect of age. Our study overcame this common limitation by including an equal stratification of young, middle, and older adults. Therefore, our observation that TMAO increases with age but is not related to cardiometabolic risk suggests that the effects of elevated TMAO may be difficult to detect in healthy populations or in early stages of cardiometabolic disease. Categorizing participants by quartiles or tertiles is commonplace in the epidemiological TMAO literature and adverse CVD outcomes are frequently reported in the highest group whose TMAO concentrations are above 5.67 [50], 6.18 [16], or 9.26 [51] μM. The fasting values observed in our cohort were much lower such that the median concentration was 3.32 μM and the threshold for the highest TMAO-tertile was 3.91 μM with a mean of 6.18 μM. Potentially, elevated TMAO concentrations coupled with an underlying cardiometabolic disease (e.g., obesity, systemic inflammation, decreased kidney function, gut microbiome dysbiosis) may dictate the physiological effect of TMAO. Furthermore, our finding that TMAO increases with age in females, but not males, supports the epidemiological observation that cardiovascular risk increases in females after menopause [52,53]. The FMO3 gene has an estrogen response element in its promoter and may influence this finding [49,54]. Future work should investigate the effects of common FMO3 single nucleotide polymorphisms in the context of pre- and post-menopause, TMAO, and CVD risk.

Our study has several limitations. Due to the study’s cross-sectional nature, we were limited to describing associations amongst the diet, microbiome, CVD risk factors, and fasting plasma TMAO. Future studies should consider a prospective design, which allow the researcher to investigate TMAO’s role in the progression from health to disease. Our study used strict exclusion criteria to enroll generally healthy individuals. However, we were unable to confirm normal blood parameters prior to enrollment, which resulted in a small number of participants with undiagnosed hyperglycemia and/or hyperlipidemia. Additionally, our food analysis was confined by the food groups categorized by the ASA24. For example, when investigating the effect of non-processed meat (defined by the ASA24 as “total of beef, veal, pork, lamb, and game meat; excludes organ meat and cured meat”), we were unable to parse the intake of one food source from the other non-processed meats. Carnitine intake is a TMAO precursor, but the nutrient has not been well characterized in foods and is not quantified by the ASA24. Furthermore, by utilizing 16s rRNA microbial sequencing, we were unable to look at the strain phylogenetic level nor were we able to draw conclusions regarding the presence of TMA generating genes.

## 5. Conclusions

Determining whether TMAO is a biomarker of concern in generally healthy individuals is an important step to discerning the clinical importance of the metabolite and to informing dietary guidelines. We report that diet components were not related to fasting plasma TMAO, that the gut microbiome has a discernable impact on TMAO, and that TMAO was not related to CVD risk factors, but was related to TNF-α in our metabolically healthy cohort of male and female adults of mixed age and BMI.

## Figures and Tables

**Table 1:** Participant characteristics by TMAO tertile.

		Tertile 1	Tertile 2	Tertile 3	*p*	Padj
	TMAO range (μM)	0.44–2.77	2.78–3.9	3.91–22.50		
Descriptive	*n*	121	120	120		
	Age (yr)	36.96 (13.43)	39.89 (13.28)	44.04 (13.93)	<0.001	0.002
	Sex (% Female)	66 (54.5)	58 (48.3)	65 (54.2)	0.557	1.0
	Ethnicity (%)				0.001	0.016
	Caucasian	67 (55.8)	71 (59.7)	82 (68.9)		
	Hispanic	9 (7.5)	21 (17.6)	19 (16.0)		
	African American	5 (4.2)	6 (5.0)	5 (4.2)		
	Asian	29 (24.2)	8 (6.7)	7 (5.9)		
	Multiple Ethnicities	7 (5.8)	10 (8.4)	4 (3.4)		
	Other	3 (2.5)	3 (2.5)	2 (1.7)		
Anthropometrics	BMI (kg/m^2^)	26.63 (4.71)	27.02 (4.70)	28.12 (5.19)	0.05	0.135
	Waist Circumference (cm)	83.10 (13.12)	84.67 (12.00)	87.58 (12.74)	0.023	0.074
Endothelial	Systolic BP (mmHg)	118.26 (10.92)	120.94 (10.39)	119.60 (11.78)	0.177	0.337
	Diastolic BP (mmHg)	68.07 (9.69)	69.54 (8.61)	67.40 (8.47)	0.169	0.337
	Reactive Hyperemia Index	2.21 (0.55)	2.26 (0.56)	2.21 (0.55)	0.768	0.768
	Augmentation Index	0.55 (21.09)	5.08 (23.92)	3.45 (21.80)	0.302	0.419
Plasma Chemistry	HDL (mg/dL)	56.72 (16.72)	55.36 (16.81)	53.48 (14.81)	0.295	0.419
	LDL (mg/dL)	106.08 (29.42)	109.56 (31.22)	114.69 (33.20)	0.101	0.2131
	Cholesterol (mg/dL)	172.11 (32.89)	174.84 (35.18)	178.10 (36.26)	0.41	0.487
	Triglycerides (mg/dL)	91.99 (47.41)	99.76 (54.33)	99.33 (40.12)	0.364	0.461
	Glucose (mg/dL)	92.33 (7.61)	95.27 (22.36)	96.52 (10.76)	0.084	0.199
	Insulin (ρM)	56.92 (36.91)	59.34 (38.17)	73.08 (75.17)	0.04	0.1210
Inflammatory	TNF-α (ρg/mL)	1.97 (0.74)	2.06 (0.82)	2.46 (0.89)	<0.001	0.000
	IL-6 (ρg/mL)	0.90 (1.94)	0.66 (0.60)	0.82 (0.57)	0.309	0.419
	CRP (mg/dL)	0.35 (0.54)	0.38 (0.84)	0.44 (0.71)	0.583	0.651
TMAO	Cystatin C (μM)	0.83 (0.12)	0.85 (0.16)	0.89 (0.13)	0.001	0.005
	TMAO (μM)	2.05 (0.55)	3.35 (0.32)	6.18 (3.11)	<0.001	0.000
	Choline (μM)	8.82 (2.05)	9.15 (2.08)	9.65 (1.93)	0.006	0.024
	Betaine (μM)	44.70 (16.69)	44.02 (15.73)	45.81 (18.64)	0.715	0.755
	Carnitine (μM)	34.80 (8.91)	35.85 (8.44)	36.77 (8.20)	0.198	0.343

Differences in the mean were tested by ANOVA and corrected for multiple testing using the Benjamini and Hochberg method. Values are mean (standard deviation) or mean (%).

**Table 2:** Relationships between fasting plasma TMAO and recent dietary intake.

		β	*p*	Padj
Protein	Non-processed Meat	0.035	0.300	0.703
	Processed Meat	0.041	0.331	0.703
	Poultry	0.025	0.458	0.734
	Seafood High in ω-3	−0.011	0.851	0.928
	Seafood Low in ω-3	0.090	0.116	0.629
	Eggs	0.035	0.582	0.764
	Nuts	0.002	0.967	0.969
	Legumes	0.021	0.631	0.764
	Total Protein Foods	0.076	0.168	0.629
Vegetables	Dark Green Vegetables	−0.126	0.060	0.629
	Red and Orange Vegetables	−0.088	0.360	0.703
	Starchy Vegetables	0.047	0.526	0.764
	Other Vegetables	−0.066	0.369	0.703
	Total Vegetables	−0.080	0.210	0.629
Grains	Whole Grains	−0.011	0.147	0.922
	Refined Grains	−0.063	0.180	0.629
	Total Grains	−0.016	0.807	0.629
Dairy	Milk	0.033	0.637	0.764
	Cheese	−0.051	0.410	0.703
	Yogurt	0.047	0.386	0.703
	Total Dairy	−0.038	0.575	0.764
Miscellaneous	Total Choline	0.111	0.208	0.629
	Total Fiber	−0.087	0.185	0.629
	HEI Total Score	<0.001	0.969	0.969

Foods were transformed to comply to the normal distribution and tested by multiple linear regression. Models were controlled for sex*age, fasting plasma Cystatin C, and total energy intake. Multiple testing was corrected via the Benjamini and Hochberg method. β represents the strength and direction of the relationship between fasting plasma TMAO and the food variable.

**Table 3:** Relationships between cardiometabolic risk factors and fasting plasma TMAO.

		β	*p*	Padj
TMAO	Betaine (μM)	−1.376	0.422	0.630
	Carnitine (μM)	1.444	0.081	0.331
	Choline (μM)	0.234	0.249	0.543
Anthropometrics	Waist circumference (cm)	1.965	0.117	0.331
	BMI (kg/m^2^)	0.829	0.100	0.331
Endothelial	Systolic BP (mmHg)	−0.233	0.838	0.950
	Diastolic BP (mmHg)	−1.756	0.054	0.305
	Reactive Hyperemia Index	−0.007	0.906	0.963
	Augmentation Index 75	−1.200	0.481	0.630
Clinical Chemistry	HDL (mg/dL)	−1.683	0.287	0.543
	LDL (mg/dL)	2.225	0.467	0.630
	Cholesterol (mg/dL)	−0.039	0.991	0.991
	Glucose (mg/dL)	0.022	0.052	0.305
	Insulin (mg/dL)	0.069	0.272	0.517
	Triglycerides (mg/dL)	0.010	0.825	0.950
Inflammatory	CRP (mg/dL)	0.129	0.326	0.554
	TNF-α (ρg/mL)	0.112	0.001	0.024
	IL-6 (ρg/mL)	0.072	0.283	0.543

Risk factor variables were transformed to comply to the normal distribution and tested by multiple linear regression. Models were controlled for sex, age, and fasting plasma cystatin C. Multiple testing was corrected via the Benjamini and Hochberg method. β represents the strength and direction of the relationship between fasting plasma TMAO and the cardiometabolic risk factor.

## Data Availability

Requests for data from the USDA-ARS WHNRC Nutritional Phenotyping Study used in this analysis should be made via an email to the senior WHNRC author on the publication of interest. Requests will be reviewed quarterly by a committee consisting of the study investigators. Scripts used in statistical analysis are available at GitHub (https://github.com/bytesizesci/FL100_TMAO, accessed on 1 February 2022).

## Supplementary Materials

The following supporting information can be downloaded at https://www.mdpi.com/article/10.3390/nu14071376/s1: Figure S1, Flow diagram of the participants in the study; Figure S2, Line plot demonstrating the sex by age interaction with respect to fasting plasma TMAO concentrations; Figure S3, Differential abundance results at the family and genus level; Table S1, Composition of standard evening meal; Table S2, Select nutrient profile of standardized evening meal; Table S3, Intake of foods by TMAO tertile; Table S4, Complete taxonomy table of the ASVs identified in the cohort; Table S5, Top ten abundant family by TMAO tertile; Table S6, Top ten abundant genera by TMAO tertile; and Table S7, Differential abundance results at the family and genus level.
