# Supplementary material for: Diet, Fecal Microbiome, and Trimethylamine N-Oxide in a Cohort of Metabolically Healthy United States Adults
Source: Nutrients. 2022 Mar 25;14(7):1376. doi: 10.3390/nu14071376 (PMC9003533; doi:10.3390/nu14071376)
Supplement: Supplementary file 1 [file nutrients-14-01376-s001.zip › nutrients-1619881-Figures.pdf]

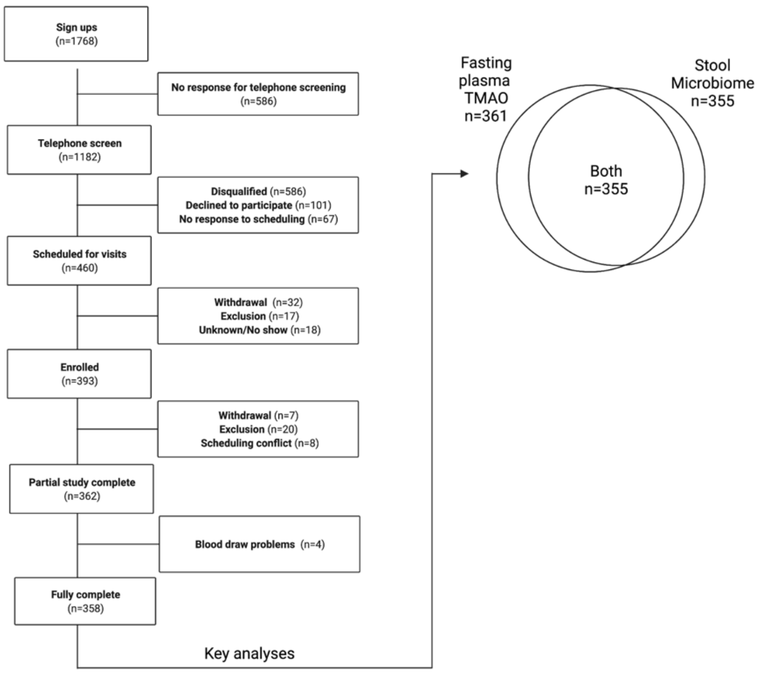

Supplemental Figure S1. Flow diagram of participants in the study and analyses.

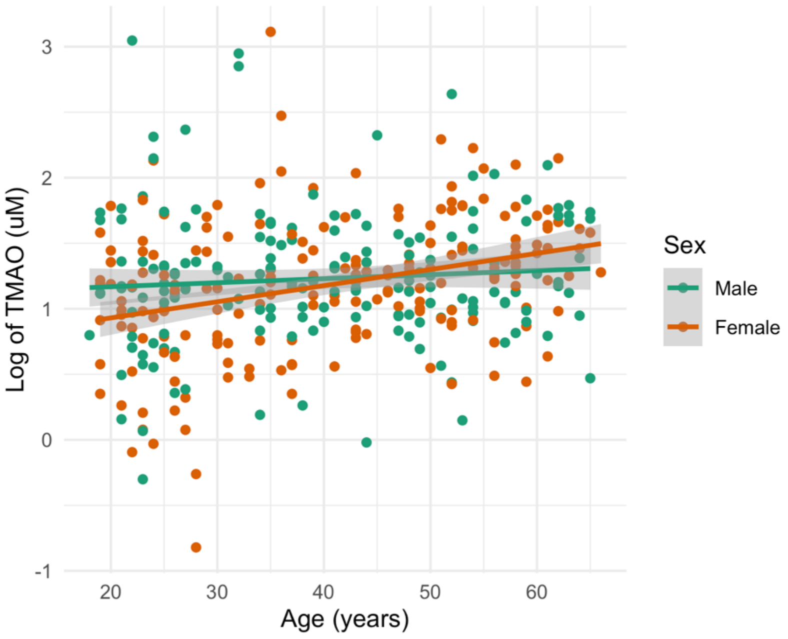

Supplemental Figure S2. A significant sex by age interaction was identified with respect to fasting plasma TMAO concentrations (P=0.026).

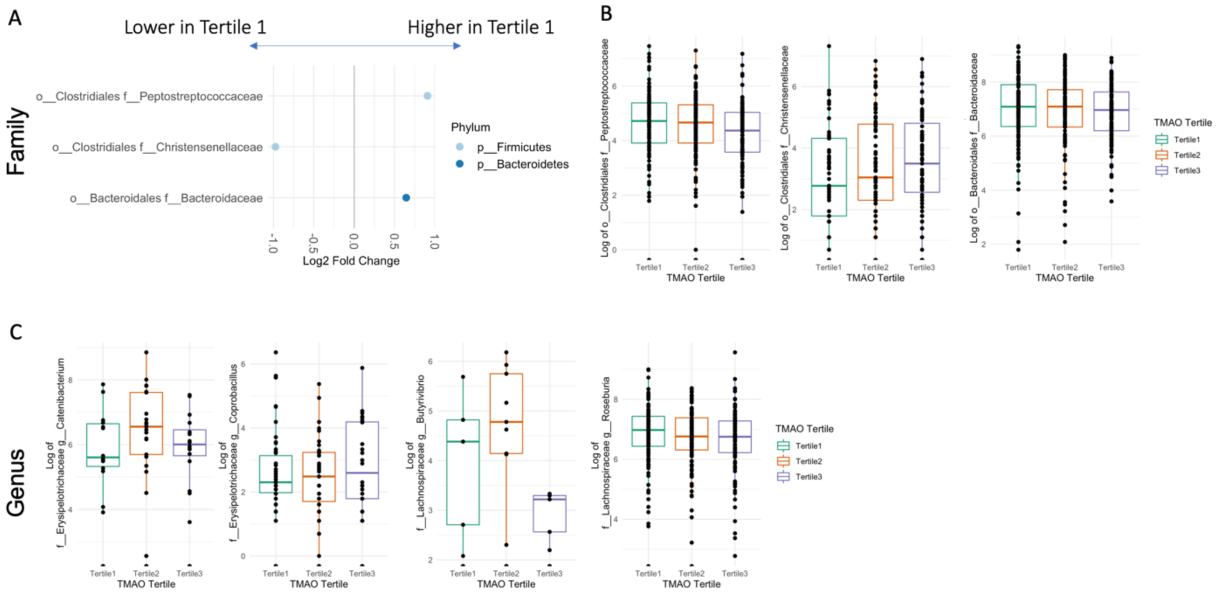

Supplemental Figure S3. (A) Family that are differentially abundant between the low (TMAO 0.44-2.77 μM) and high TMAO tertile (TMAO 3.91-22.5 μM). The graph is relative to the low TMAO group such that points to the right of 0 are more abundant in participants with the lowest TMAO values. Distributions of the differentially abundant taxa by TMAO tertile at the (B) family and (C) genus levels.
